# Efficacy of Perampanel in Refractory and Super-Refractory Status Epilepticus with Suspected Inflammatory Etiology: A Case Series

**DOI:** 10.3390/ph17010028

**Published:** 2023-12-24

**Authors:** Annacarmen Nilo, Alberto Vogrig, Marco Belluzzo, Christian Lettieri, Lorenzo Verriello, Mariarosaria Valente, Giada Pauletto

**Affiliations:** 1 Clinical Neurology Unit, Department of Head, Neck and Neurosciences, Santa Maria della Misericordia University Hospital, 33100 Udine, Italy; alberto.vogrig@gmail.com (A.V.); christian.lettieri@asufc.sanita.fvg.it (C.L.); mariarosaria.valente@uniud.it (M.V.); 2 Department of Medicine, University of Udine, 33100 Udine, Italy; 3 Neurology Unit, Department of Head, Neck and Neurosciences, Santa Maria della Misericordia University Hospital, 33100 Udine, Italy; marco.belluzzo@asufc.sanita.fvg.it (M.B.); lorenzo.verriello@asufc.sanita.fvg.it (L.V.); giada.pauletto@asufc.sanita.fvg.it (G.P.)

**Keywords:** status epilepticus, perampanel, neuroinflammation, treatment, retrospective study

## Abstract

(1) Background: Increasing evidence supports the anti-inflammatory and neuroprotective role of perampanel (PER), mediated by decreased expression of pro-inflammatory cytokines and by interference with apoptosis processes. Therefore, the use of PER to treat status epilepticus (SE) with suspected inflammatory etiology is appealing and deserves further investigation. (2) Methods: We retrospectively analyzed seven patients (five F, two M; median age: 62 years) with refractory and super-refractory SE due to a probable or defined inflammatory etiology and treated with PER. (3) Results: PER was administered as the third (4/7) or fourth drug (3/7), with a median loading dose of 32 mg/day (range: 16–36 mg/day) and a median maintenance dose of 10 mg/day (range: 4–12 mg/day). In five cases, SE was focal, while in two patients, it was generalized. SE was caused by systemic inflammation in three patients, while in the other four subjects, it was recognized to have an autoimmune etiology. SE resolution was observed after PER administration in all cases, particularly within 24 h in the majority of patients (4/7, 57.1%). (4) Conclusions: Our data support the efficacy of PER in treating SE when first- and second-line ASMs have failed and suggest a possible earlier use in SE cases that are due to inflammatory/autoimmune etiology.

## 1. Introduction

Status epilepticus (SE) is a life-threatening condition and a neurological emergency with high morbidity and mortality (from 10% of cases up to 50% in older patients) [1,2,3]. According to the definition from the International League Against Epilepsy (ILAE), SE instauration derives from the failure of the mechanisms responsible for seizure termination or from the development of mechanisms that lead to abnormally prolonged seizures (after time point t_1_) [1]. The persistence of continuous seizure activity can have long-term consequences (after time point t_2_), including neuronal injury and death and alteration of neuronal networks [1]. When SE does not respond to first- or second-line medications, it is considered refractory (RSE, refractory status epilepticus) and requires treatment with systemic anesthetics [4]. Furthermore, if RSE persists despite treatment with anesthetics for at least 24 h, it is referred to as super-refractory SE (SRSE) [4].

It has been hypothesized that glutamate (Glu) can play a fundamental role in triggering and sustaining epileptic firing through its excitatory activity exerted on the ionotropic alpha-amino-3-hydroxy-5-methyl-4-isoxa-zol-propionic (AMPA) receptors [5]. In fact, Glu is the main excitatory neurotransmitter, and glutamate-mediated hyperexcitability represents one of the most important epileptogenic mechanisms. The imbalance of inhibitory gamma-aminobutyric acid (GABA) and excitatory Glu plays a crucial role in the development and persistence of refractory SE [6]. Changes in receptor density and in expression on the cell surface, with the internalization of synaptic GABA_A_ receptors, the upregulation of extra-synaptic GABA receptors, and the accumulation of excitatory glutamatergic receptors on the neuronal surface, contribute to increasing and perpetuating a state of excitability [6]. Moreover, it has been recently described that Glu also plays a role as a gliotransmitter. Released by glial vesicles, Glu modulates synaptic efficiency and the release of pro-inflammatory cytokines [7].

Perampanel (PER) is the first drug specifically designed to modulate AMPA receptors, and it is a third-generation anti-seizure medication (ASM). In fact, it is an active, noncompetitive, selective antagonist of AMPA receptors, specifically engineered to block the glutamate activity at post-synaptic receptors [8]. It is taken orally in a single dose at bedtime, and it is available as tablets and oral suspensions; thus, it can also be administrated via a nasogastric tube. Perampanel is rapidly and completely absorbed after oral administration [8]. It is metabolized essentially by the liver via CYP3A4/5, but it does not exert any significant inhibitory or inductive effects on liver metabolism [8]. Therapeutic doses range from 4 to 12 mg/day, even though in selected patients, clinical benefits could already be observed at 2 mg/day. PER has been approved [9] as an adjunctive therapy for the treatment of focal seizures with or without secondary generalization in patients aged ≥4 years and as an adjunctive treatment of primary generalized tonic–clonic seizures (GTCSs) associated with idiopathic generalized epilepsy in patients ≥ 12 years (≥7 years in the EU) [9,10], based on the results derived from four randomized controlled trials (RCTs). In the USA only, the drug has also received authorization as monotherapy [9]. PER has demonstrated a good safety profile, and it does not interact with other ASMs, favoring the possibility of a rational and unrestricted add-on therapy. Interactions with other kinds of medication are limited and generally not clinically relevant [8]. It is generally well-tolerated, reporting only mild or moderate adverse events (AEs). In particular, dizziness was the most frequent side effect, followed by somnolence, fatigue, irritability, weight increase, headache, ataxia, and falls [8]. Real-world data have confirmed the good tolerability of perampanel, even in special populations such as the elderly [11], patients with tumor-related epilepsy [12], and subjects with highly pharmacoresistant epilepsy [13].

Studies on seizure models such as those of traumatic brain injuries (TBIs) and pilocarpine–lithium rat models have demonstrated that perampanel may reduce apoptosis and decrease the synthesis of cytokines such as TNF-α, IL-1ß, IL-6, and IL-10 as well as inducible nitric oxide synthetase (iNOS) and neuronal NOS (nNOS) [14,15]. Taken together, this evidence suggests the possible use of PER in mitigating seizure risk in brain inflammation, as in cases of SE. Although inflammatory response is common in all RSE and SRSE cases, SE with inflammatory etiology may benefit more from early use of PER.

The present study aimed at retrospectively evaluating the efficacy of PER in a cohort of patients with refractory and super-refractory SE with definite and/or probable inflammatory etiology.

## 2. Results

Over the study period (January 2019–December 2021), 70 patients were treated for SE in our department (Santa Maria della Misericordia University Hospital of Udine, Udine, Italy); among them, 15 were diagnosed with SE due to inflammatory etiology, but only 7 completely met the inclusion criteria. Two patients were males (29%) and five females (71%), with a median age of 62 years (range: 42–91 years). The majority of them recognized an autoimmune etiology of the SE (4/7, 57%). Particularly, two cases were diagnosed with a New Onset of Refractory Status Epilepticus (NORSE) (patients 5 and 6), one had a reactivation of multiple sclerosis (MS) (patient 4), and one was affected by Systemic Lupus Erythematosus (SLE) with central nervous system (CNS) involvement (patient 1).

The patients with NORSE underwent an extensive and comprehensive diagnostic work-up consisting of blood tests, lumbar puncture, and brain Magnetic Resonance Imaging (MRI), which was unremarkable. Other etiologies were excluded; thus, a possible seronegative dysimmune etiology was hypothesized. The remaining three cases (43%) presented with SE during acute systemic inflammatory conditions; two subjects had SARS-CoV-2 infection (patients 2 and 7), and one was diagnosed with sepsis-related Systemic Inflammatory Response Syndrome (SIRS) (patient 3). In these patients, other etiologies were excluded, as per routine clinical practice, and the typical blood inflammatory parameters (C-reactive protein, procalcitonin, and cytokines, when available) showed increased values.

Regarding SE semiology, we identified two focal non-convulsive SE (NCSE) cases with aphasic manifestations (patients 1 and 4), three focal motor SE cases without impairment of awareness (one clonic, one tonic, and one inhibitory, also known as ictal paresis: patients 5, 3, and 2, respectively), and two generalized NCSE cases with coma (patients 6 and 7). Status epilepticus lasted a mean of 65.4 h (range: 48–96 h). Since the onset of SE, all patients underwent continuous electroencephalogram (cEEG) monitoring.

Perampanel was used as a third medication in four of seven patients (57%) and as a fourth medication in the remaining three (43%). A median of 2.4 previous ASMs was administrated without clinical benefits. In all patients, the baseline Status Epilepticus Severity Score (STESS) was calculated, indicating the severity of the SE: the median STESS was equal to 2.1 (range: 0–5). Three patients, specifically those with STESS ≥ 3, required Intensive Care Unit (ICU) admission before starting PER.

**Figure 1:**
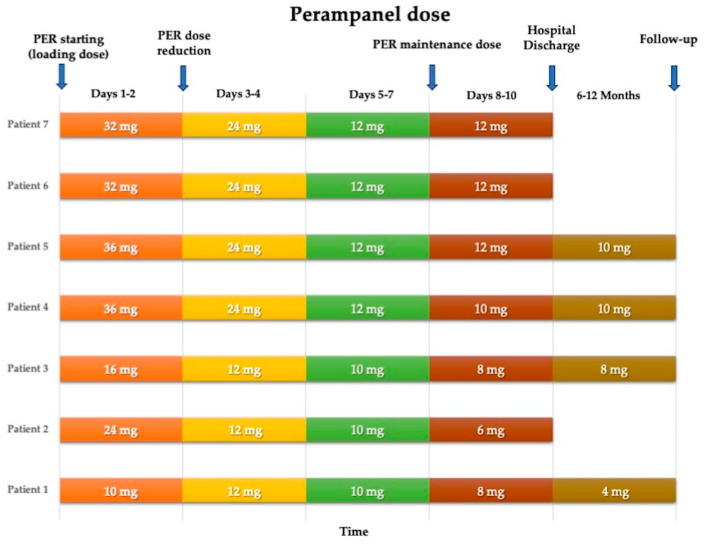
Perampanel dose reduction over time for each patient.

The drug was administered orally, when needed, via a nasogastric tube as an oral suspension, with a loading dose and a subsequent maintenance dose. The median loading dose was 32 mg/day (range: 16–36 mg/day), with a median maintenance dose of 10 mg/day (range: 4–12 mg/day). The maintenance dose was achieved by progressively reducing the daily dose over 2 or 3 days from the start (Figure 1).

The administration of PER resulted in the resolution of both clinical and electrical SE in all seven patients, within a median of 34.3 h from drug administration. In four cases (patients 1, 2, 3, and 4), SE resolution was obtained within 24 h, and in three patients (patients 5, 6, and 7) within 48 h from the start of PER, regardless of whether or not the underlying disease had improved. The time from SE diagnosis to the first PER administration was extremely variable, from 24 to 48 h (median time: 41.1 h).

Three patients died during hospitalization. Death was due to etiologies other than SE: two patients died from pulmonary embolism, and one from severe respiratory failure secondary to SARS-CoV-2 infection. At hospital discharge, all four living patients continued the treatment with PER.

Seizure outcome was assessed 6 and/or 12 months after the resolution of SE and therefore from the start of PER. Three subjects were seizure-free, while the remaining one had only sporadic auras. The median dose of PER, at follow-up, was 8 mg/day (range: 4–10 mg/day).

Regarding safety concerns, treatment-emergent AEs were observed only in one patient, in the acute phase, consisting of a transient increase in liver enzymes, which was observed after loading dose and spontaneously regressed in a few days. Subsequently, the surviving patients reported neither daytime sleepiness nor dizziness or behavioral or mood changes.

The main clinical features of the seven patients are shown in Table 1.

## 3. Discussion

Clinical studies investigating the efficacy of PER in the treatment of RSE and SRSE are limited. Most data come from real-life experiences on single cases or series with widely varying evidence of effectiveness and tolerability [16,17,18,19,20].

To date, only four retrospective cohort studies have been completed [21,22,23,24]. However, the interpretation of the effect of PER derived from these reports should be weighted carefully due to the variability in criteria used to measure efficacy, differences in latency from SE onset to PER administration, and, most of all, the heterogeneous etiology of the SE in the cohorts’ studies.

According to many authors, the underlying etiology of SE is one of the main predictors of the response to pharmacological treatment [25,26]. Consequently, there is a need to evaluate and validate the efficacy of ASMs in specific sub-categories of SE based on the underlying etiology in order to guarantee targeted and personalized therapy. Moreover, the use of anesthetic drugs carries potentially life-treating risks, such as respiratory failure and cardiac dysfunction, which may require additional invasive treatments, such as ventilation and intravenous catecholamines, so they should be used cautiously, in particular in complex and frail patients, such as subjects with RSE and SRSE [27]. 

It is established that AMPA receptors play a relevant role during and after SE. In the CNS, Glu serves as the primary excitatory neurotransmitter, and N-methyl-D-aspartate (NMDA) and AMPA receptors are widely distributed [28]. It is known that the current paradigm of the SE entails the downregulation of GABA_A_ receptors and the upregulation of NMDA and AMPA receptors. Notably, these two glutamatergic receptors play a crucial role in regulating ion permeability, particularly Ca^2+^, and may contribute to the initiation and maintenance of seizures and SE [28]. As indicated by preclinical data [29], the AMPA receptor antagonists present a potential therapeutic effect for patients with SE, especially in the early phase. Given that some autoimmune encephalitides involve antigens targeting these neuronal surface receptors (including AMPA and NMDA receptors), leading to seizures resistant to ASMs [30], exploring the impact of pharmacological receptor blocking and mitigating symptoms in such cases becomes particularly intriguing. Interestingly, isolated reports suggest the beneficial effects of perampanel in AMPA receptor encephalitis [31]. In this condition, autoantibodies target a neuronal surface protein, resulting in a limbic encephalitis phenotype characterized by seizures, confusion, and anterograde memory dysfunction. Similarly, a positive effect has been observed in anti-NMDA receptor encephalitis [32], implying that the impact may be related to a broader downregulation of the excitatory neurotransmitter glutamate rather than a receptor-specific effect. This observation paves the way for future studies investigating the role of PER in autoimmune encephalitis-related seizures and SE.

As reported in preclinical studies, PER has been shown to terminate benzodiazepine-resistant SE in a lithium pilocarpine rat model after 30 min at a median dose of 5.1 mg/kg^−1^ [33]. Successively, data in humans have supported the use of AMPA receptor antagonists, such as PER, in the treatment of refractory SE [29].

Moreover, increasing evidence is emerging regarding the anti-inflammatory and neuroprotective role of PER, mediated by decreased expression of pro-inflammatory cytokines and interference with apoptosis processes [14,34].

The neuroinflammatory response is a necessary process that is initiated immediately after a brain insult and may be partly common for different etiologies of SE.

Therefore, the use of PER in the treatment of SE, particularly in the case of SE due to inflammatory etiology, finds its own rationale.

In our population, we described the effect of PER to resolve RSE within 24 and 48 h after its administration in 4/7 and 3/7 patients. In all patients, PER was the last administrated drug after a median of 2.4 previous ASMs. PER was effective both in motor and non-motor SE. Considering SE clinical characteristics, focal SE was demonstrated to respond faster after PER administration compared to generalized SE. Particularly, in our series, patients 1 and 4 with focal NCSE with aphasia and patients 2 and 3 with focal inhibitory and tonic SE recovered from SE, 24 h after starting PER. In our series, no severe AEs were reported. In one patient only, there was a transient increase in transaminase blood levels after loading dose, which spontaneously regressed in a few days. Other authors have reported the same transient increase in liver enzymes [18,35]. Interestingly, no psychiatric side effects have been observed, even if some patients were elderly (patients 2, 5, and 6) and some underwent general anesthesia with consequent permanence in the ICU, which is a clinical setting predisposing for delirium and behavioral changes (patients 5, 6, and 7). Similarly, psychiatric or behavioral side effects were not reported in the other cohorts [16,17,18,23,24].

In the literature, PER has been evaluated in the treatment of SE with different etiologies. Thus, the range of responders spans from 16% [18] to 75% [16,36], depending on the underlying SE causes [23]. Furthermore, different treatment choices and timing of ASMs administration increase the heterogeneity of the results and prevent generalizable conclusions.

Lim et al. [23] assessed the efficacy of PER in treating RSE and SRSE in a large cohort of 81 patients. Perampanel determined SE resolution in 33.3% of cases with a potential correlation with the initial PER dose and the timing of PER administration from SE onset. The authors observed that a higher responder rate was achieved in NCSE and in acute symptomatic SE (such as SE due to brain tumor, hypoxic damage, CNS, and systemic infections) [23]. They highlighted the importance of etiology in choosing the appropriate ASM and the timing of administration. A satisfactory safety profile was confirmed; in fact, no cardiorespiratory AEs or laboratory abnormalities were reported [23]. Similarly, in their retrospective cohort study, Ho et al. [24] observed that PER was effective in 36.4% of SE cases within 4 days from the first drug administration. In particular, they reported a greater efficacy of PER at lower doses in patients with focal motor and generalized convulsive SE rather than subjects with NCSE [24]. In another work, Rohracher et al. analyzed the clinical outcomes of 30 patients with RSE and SRSE, mainly NCSE with coma, who were treated with PER at different doses (from 4 mg/day to 32 mg/day) [18]. The underlying causes of SE were heterogeneous, with five patients out of 30 with CNS infections and no one with possible or definite autoimmune etiologies [18]. Globally, the clinical benefit of PER was observed only in 17% of patients. Noteworthly, the authors did not find differences in treatment response, clinical outcome, or time to ICU discharge when comparing low doses of PER (eventually up-titrated to 2 mg daily) with the higher dose loading of 32 mg/day [18]. In critically ill patients, severely impaired gastrointestinal absorption is not improbable, which may compromise PER absorption and, thus, its efficacy [18]. This represents an aspect that should be taken into account when clinicians choose the type and the dose of oral ASMs, such as PER. Similarly to our findings, they did not observe significant AEs, even when PER was administrated at high doses [18]. In another Italian study, the efficacy of PER was evaluated in patients with post-anoxic refractory NCSE [16]. Eight post-anoxic patients with super-refractory NCSE and good prognostic indicators were treated with PER, with a resolution of SE within 72 h in six subjects (75%). Furthermore, half of responder patients showed a good neurological outcome at three months from hospital discharge. Regarding safety, the authors observed a mild cholestatic injury after starting PER in five patients, which spontaneously resolved without clinical sequelae [16].

Considering the significant prognostic role of SE etiology, we focused on inflammatory forms of SE. It is known that CNS or severe systemic infections and defined or possible autoimmune etiologies may account for a large proportion of SE cases. Moreover, an inflammatory origin may be due to still-unknown autoantibodies in a subset of patients. SE itself causes brain inflammation, promoting self-sustaining epileptic circuits and neuronal death or damage [37,38]. In fact, in temporal sclerosis secondary to SE, activation of microglia, glial and neuronal upregulation of IL-1ß, high mobility group box 1 (HMGB1), COX-2, complement system, and downstream inflammatory mediators have been described [39,40,41]. Extensive extravasation of albumin into the brain of patients who died of SE has been reported, indicating a massive alteration of the brain–blood barrier (BBB). Studies on different animal models of SE demonstrated an intensive inflammatory cascade in the forebrain. Increasing IL-1ß, TNF-α, and IL-6 transcript levels are observed 30–60 min after the onset of SE in the hippocampus or forebrain, associated with increased neuronal COX-2 protein [38,42,43,44]. Activation of microglia, reactive gliosis, and infiltration by monocytes follow shortly afterward [38,45].

A concomitant inefficient anti-inflammatory endogenous control has also been advocated in igniting and maintaining SE [39,46,47]. Furthermore, in animal models, pharmacological trials with drugs targeting inflammatory pathways during SE were able to reduce the severity of subsequent epilepsy [38].

There have been different attempts to damp the inflammatory response during RSE and SRSE, from the common use of glucocorticoids to anakinra in the case of Febrile Infection-Related Epilepsy Syndrome (FIRES) [48].

Recently, it has been proposed that perampanel may play a role in modulating inflammatory cytokine release and interfering with apoptotic processes [14,34].

AMPA receptors are composed of four sub-units (from GluA1 to GluA4) and can be either homo- or hetero-tetrameric [7]. Each subunit has different characteristics. Specifically, GluA2 is a determinant of calcium permeability. In particular, the expression of AMPA calcium permeable receptors in excitatory synapses increases both in physiological and pathological conditions due to the mechanism of synapse plasticity. Moreover, AMPA receptors are expressed in glial cells, and inflammation may determine the hyper-expression of AMPA receptors in astrocytes, inducing excitotoxicity [5]. Microglia and macrophages express AMPA receptors whose activation contributes to the release of pro-inflammatory cytokines [5]. The over-expression of AMPA receptors, which can occur in several conditions, including SE, seems to determine, at the level of microglia and astrocyte cells, an early and increased expression of genes that regulate the production of pro-inflammatory cytokines, particularly TNF-α [49]. Therefore, the use of AMPA receptor antagonists, such as PER, may interfere with this process, as shown by recent studies in animal models [14,34]. Accordingly, the administration of PER in the lithium-pilocarpine SE rat model determined not only the cessation of seizures but also a neuroprotective action preventing neuronal damage [14,50]. Furthermore, in studies on the TBI model in rats, PER administration suppressed the expression of pro-inflammatory cytokines TNF-α and IL-1ß but increased the levels of anti-inflammatory cytokines IL-10 and TGF-ß1 at 4 and 10 h after TBI, showing a potential anti-oxidative and anti-inflammatory role [34].

These preclinical studies support the use of PER in clinical conditions where inflammation is predominant, such as SE. Thus, taking thoroughly into account both pre-clinical data and clinical experiences (even with all the bias of real-world data and complex clinical settings), PER could be an option to shorten SE, particularly by decreasing the inflammatory response as well as by acting with its unique mechanism of action.

## 4. Materials and Methods

This is a retrospective, monocentric, observational study of patients with RSE and SRSE recognizing a possible inflammatory etiology treated with PER.

All patients were admitted to the Neurology Unit and the Clinical Neurology Unit of Santa Maria della Misericordia University Hospital between 1 January 2019 and 31 December 2021.

SE was defined and classified according to the latest classification proposed by the ILAE [1]. SE was defined as “refractory” following the failure of first- and second-line therapies when the use of deep sedation was required; it was defined as “super-refractory” when it did not recede despite 24 h or more of general anesthesia or if it recurred on sedation withdrawal [1].

SE has been classified as having inflammatory etiology in the following cases:

- Patients with known autoimmune disease and symptomatic epilepsy (in the absence of other causes of SE);
- Patient with definite autoimmune encephalitis according to current diagnostic criteria [51,52];
- Patients with a systemic inflammatory state based on laboratory and clinical parameters;
- Patients with NORSE, when other causes of SE have been excluded.

Therefore, the following inclusion criteria were applied:

- Age ≥ 18 years;
- Diagnosis of RSE or SRSE with definite or possible inflammatory etiology;
- Availability of continuous electroencephalographic (c-EEG) monitoring from the diagnosis to the resolution of SE;
- Use of PER as treatment during SE;
- Presence of complete clinical, laboratory, and instrumental data;
- Clinical follow-up of at least 6 months in SE survivors.

Clinical data were retrieved from medical charts. The following variables were collected: demographic data (gender, age); previous history of epilepsy (with seizure type and frequency and treatments) or previous SE; SE type (focal/generalized; convulsive/nonconvulsive), etiology and duration; blood and/or cerebrospinal fluid (CSF) parameters; type and number of ASMs and other concomitant therapies; timing of PER use; loading dose and maintenance dose; time from PER administration to SE resolution; need for deep sedation; EEG pattern; adverse events related to PER; SE outcome and mortality; persistence of seizures at 6 and/or 12 months after SE resolution.

To assess the short-term prognosis of SE, STESS [53] was calculated for every patient at baseline.

The choice regarding second-line anti-seizure therapy followed the Italian League Against Epilepsy (Lega Italiana Contro l’Epilessia, LICE) guidelines [54], while the choice of third-line therapy was based on the current clinical practice in our ICUs.

c-EEG monitoring was available for all patients. The c-EEG monitoring was performed according to the 10–20 International System by means of SystemPLUS EVOLUTION software, version 2016 (Micromed^TM^, Treviso, Italy). EEG traces were categorized based on the recorded activity, as suggested by the Salzburg Criteria [55]. Each EEG trace was blindly reviewed off-line by two experienced neurophysiologists (A.N. and G.P.). In case of discrepancy, a final review was performed by a third neurophysiologist (C.L.).

### 4.1. Study Outcomes

We considered a trial with PER successful in terminating SE (effectiveness of PER) when it was: (1) the last drug administered within 72 h prior to the clinical and/or EEG resolution of SE, without changes in other concomitant medications, and (2) SE did not recur during the entire hospital observation of the patient, using previously adopted definitions [56,57].

Secondary outcomes were the incidence and features of AEs observed for PER. We classified an event as “adverse event” if it appeared in close temporal relationship with the administration of PER and if it is reported in the safety profile of the drug.

The response to treatment was monitored clinically and with EEG to verify the disappearance of continuous epileptic activity.

Finally, we evaluated seizure frequency at 6 and/or 12 months after discharge.

The Institutional Review Board (I.R.B.) of the University of Udine approved the investigation (protocol: 117/2022 Tit III cl 13 fasc.8/2022).

### 4.2. Statistical Analysis

The collected data were reported in an anonymized database created ad hoc in Excel 2022 (Microsoft Corp., Redmond, WA, USA). The descriptive analysis was performed by means of centrality measures such as mean values ± standard deviation or median values and range for continuous variables and by means of percentages for categorical variables. All statistical analyses were performed using SPSS version 25.0 (IBM Corporation, Chicago, IL, USA).

## 5. Limitations

Our study is a preliminary report, and it is fraught with several limitations, such as the retrospective observational design, the small number of cases, and the heterogeneity of inflammatory etiology. In addition, the use and dose of PER were at the discretion of the treating physician, based on personal experience, which may under- or over-estimate the treatment response to PER due to the selection of patients who received the drug. Furthermore, it is difficult to separate the effect of PER from the concomitant use of other ASMs, the type of sedation, or other concurrent medications. In addition, we cannot rule out the possibility of spontaneous resolution of the underlying cause.

## 6. Conclusions

Perampanel was successful in terminating SE without significant adverse events in our cohort of complex patients with refractory forms of SE. Our case series, albeit small, broadens the clinical experiences on the efficacy and safety of PER as a treatment for SE when first- and second-line ASMs have failed. It may also support the early use of PER in the case of inflammatory SE, considering the role of AMPA receptors and Glu in sustaining epileptic firing and promoting inflammatory cytokine release by microglia. Larger prospective studies, possibly with standardized treatment and dosing protocol, are needed to confirm these preliminary observations.

## Figures and Tables

**Table 1:** Clinical features of patients with status epilepticus with inflammatory/autoimmune etiology treated with perampanel.

Patient	Age/Gender	Epilepsy History	SE Type	SE Etiology	STESS	Previous ASMs	Other Treatments	ICUs Access	Time to SE Onset and PER Administration	Total SE Duration	Time between PER Administration and SE Resolution	Death	Epilepsy’s Outcome at 6–12 Months
Nr. 1	49 yrs/F	Yes	Focal NCSE with aphasia	Autoimmune SLE	1	2 (LEV, LCM)	No	No	48 h	72 h	24 h	No	Seizure-free
Nr. 2	91 yrs/F	Yes	Focal inhibitory SE	Systemic infection due to SARS-CoV-2	2	3 (LEV, LCM, VPA)	No	No	48 h	72 h	24 h	Yes (pulmonary embolism)	-
Nr. 3	42 yrs/F	Yes	Focal tonic SE	Systemic bacterial sepsis	0	2 (LEV, LCM)	No	No	24 h	48 h	24 h	No	Seizure-free
Nr. 4	61 yrs/F	Yes	Focal NCSE with aphasia	Autoimmune: multiple sclerosis reactivation	0	2 (LEV, VPA)	Steroids	No	48 h	72 h	24 h	No	Only some auras
Nr. 5	70 yrs/M	No	Focal clonic motor SE	NORSE of presumed autoimmune etiology	3	2 (LEV, LCM)	Steroids, intravenous immunoglobulin	Yes	48 h	96 h	48 h	No	Seizure-free
Nr. 6	70 yrs/M	No	Generalized NCSE with coma	NORSE of presumed autoimmune etiology	5	2 (LEV, LCM)	Intravenous immunoglobulin	Yes	48 h	96 h	48 h	Yes (acute respiratory failure)	-
Nr. 7	62 yrs/F	No	Generalized NCSE with coma	Systemic infection due to SARS-CoV-2	4	3 (LEV, LCM, VPA)	No	Yes	24 h	72 h	48 h	Yes (pulmonary embolism)	-

Abbreviations: ASMs, anti-seizure medications; h, hours; LCM, Lacosamide; LEV, Levetiracetam; NCSE, non-convulsive status epilepticus; Nr, number; SE, status epilepticus; STESS, status epilepticus severity score; VPA, Valproic Acid; yrs, years.

## Data Availability

Data Access, Responsibility, and Analysis: all authors have full access to all the data in the study and take responsibility for the integrity of the data and the accuracy of the data analysis.
